# Supplementary material for: Testing the intrinsic mechanisms driving the dynamics of Ross River Virus across Australia
Source: PLoS Pathog. 2024 Feb 15;20(2):e1011944. doi: 10.1371/journal.ppat.1011944 (PMC10868856; doi:10.1371/journal.ppat.1011944)
Supplement: S1 Appendix — Mosquito population trends by mosquito species and study site. (DOCX) [file ppat.1011944.s001.docx]

S1 Appendix – Vector population abundance over time per site


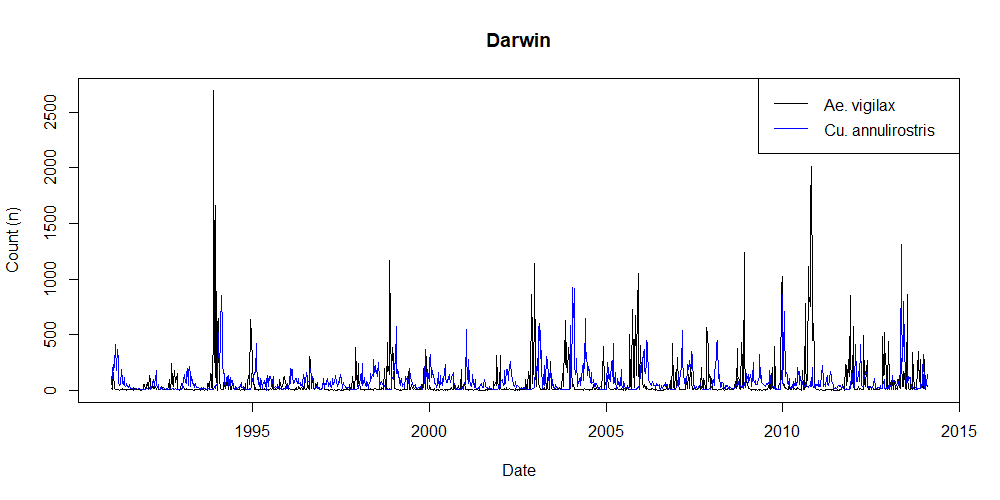
Appendix Figure 1 – Vector species population trend in Darwin.


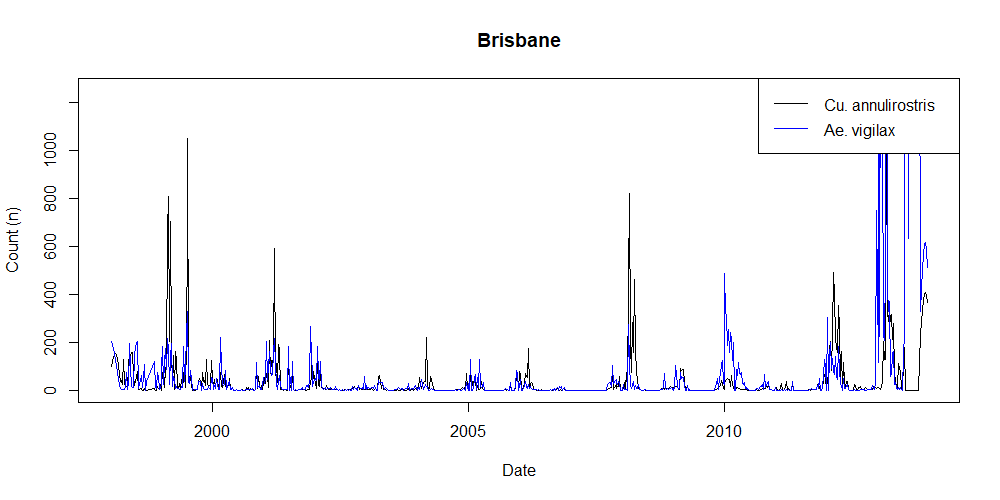
Appendix Figure 2 – Vector species population trend in Brisbane.


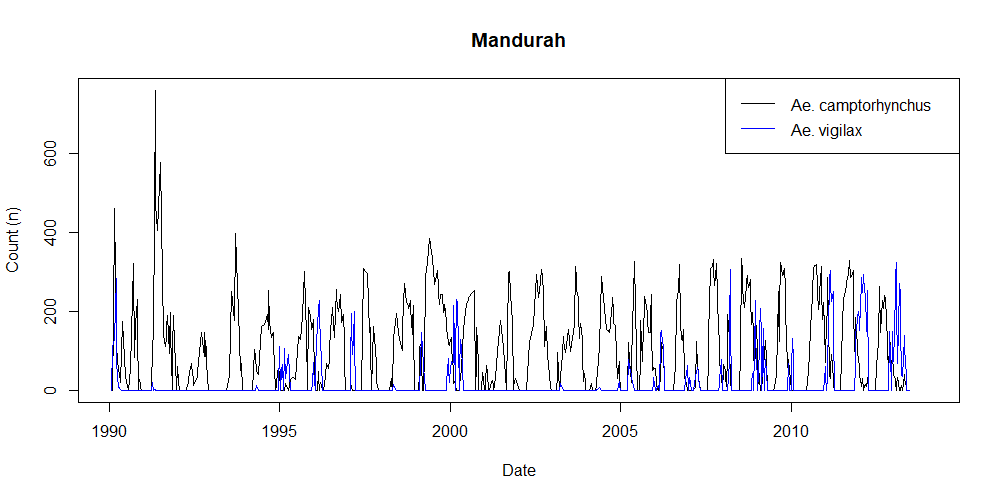
Appendix Figure 3 – Vector species population trend in Mandurah.


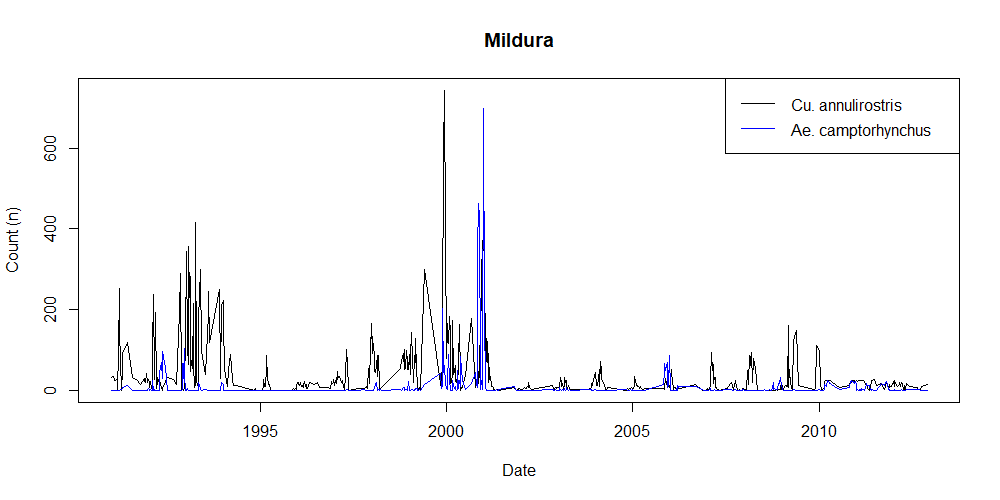
Appendix Figure 4 – Vector species population trend in Mildura.


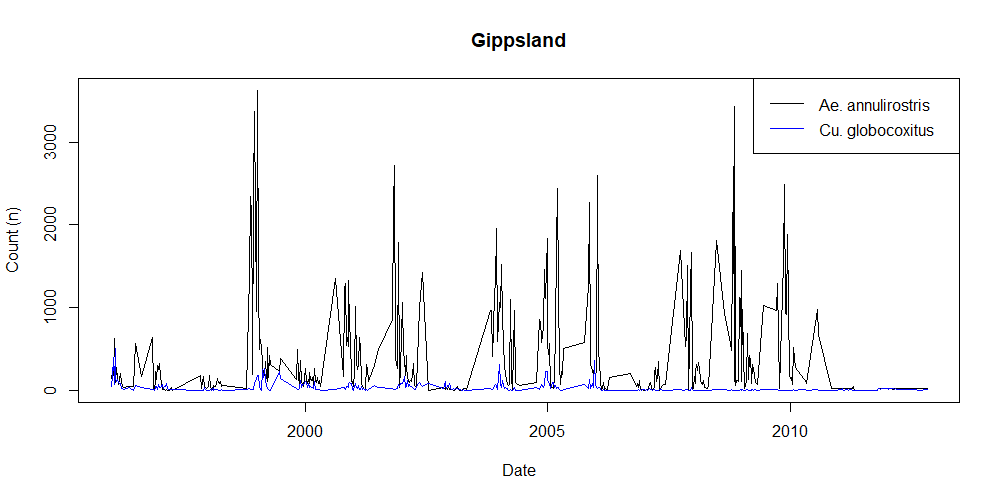
Appendix Figure 5 – Vector species population trend in Gippsland.


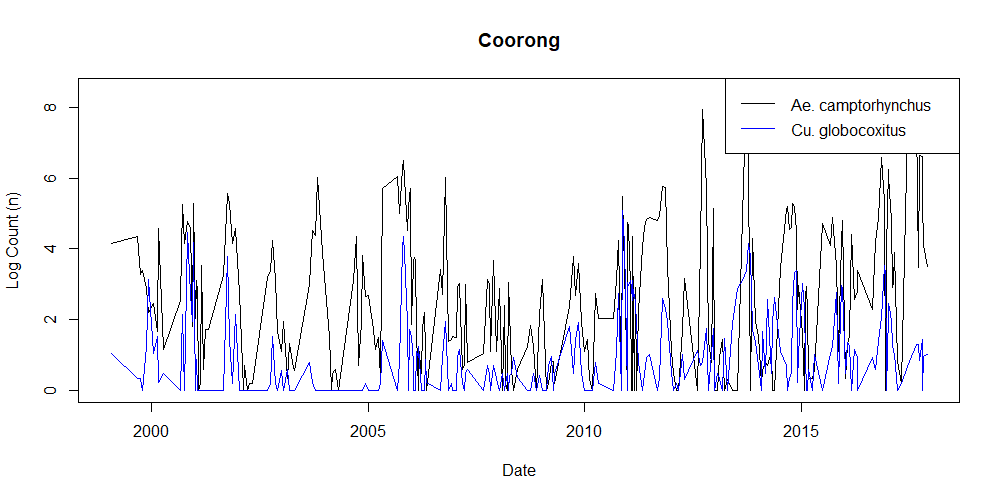
Appendix Figure 6 – Vector species population trend in Coorong.


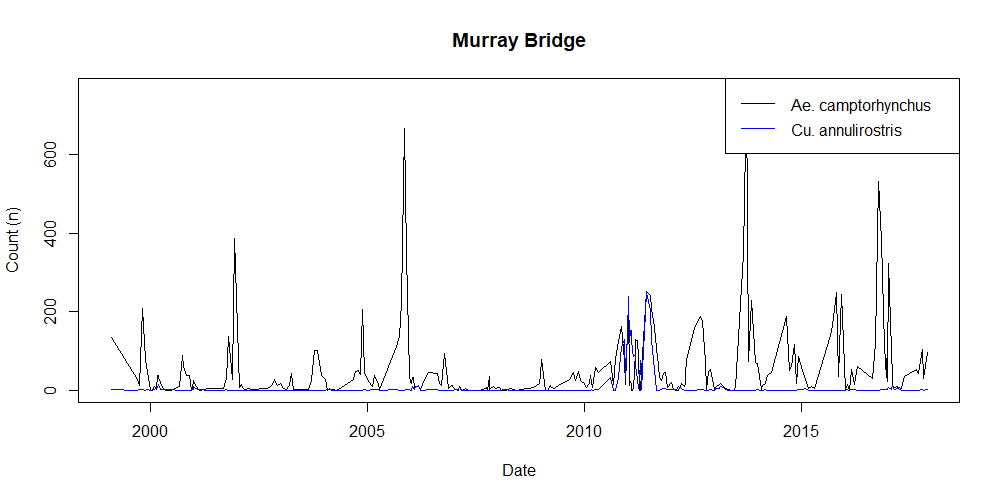
Appendix Figure 7 – Vector species population trend in Murray Bridge.


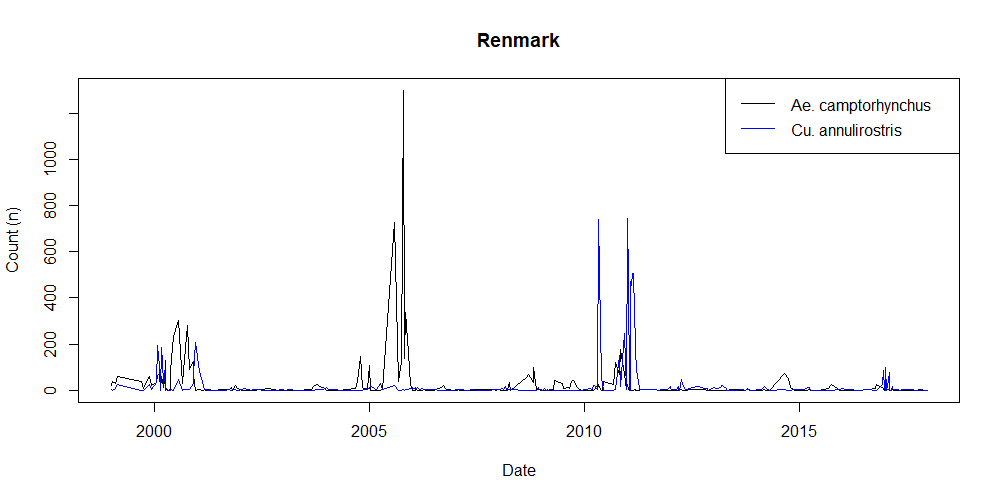
Appendix Figure 8 – Vector species population trend in Renmark.
