# Supplementary material for: Testing the intrinsic mechanisms driving the dynamics of Ross River Virus across Australia
Source: PLoS Pathog. 2024 Feb 15;20(2):e1011944. doi: 10.1371/journal.ppat.1011944 (PMC10868856; doi:10.1371/journal.ppat.1011944)
Supplement: S2 Appendix — Code and solver used to run the ordinary differential equations. (DOCX) [file ppat.1011944.s002.docx]

S2 Appendix – Fortran and R code

Fortran solver code:

subroutine initmod(odeparms)

external odeparms

double precision, dimension(1499) :: bh

double precision, dimension(1500) :: years, nm, nm2

double precision, dimension(1500) :: bm,bm2,bk,bp,Nm_mod,Nm2_mod,Nk,Np,Nh_mod

double precision parms(26)

common /myparms/parms

common /data/years,nm,nm2,bh,n

common /derived/bm,bm2,bk,bp,Nm_mod,Nm2_mod,Nk,Np,Nh_mod

! common /myparms/dens1,gamma_h2,scale_m3,Nm_min4,Nm2_min5,v6,&

! gamma_k7,N_K_med8,mu_k9,delta_k10,theta_k11,omega_k12,gamma_p13,&

!N_P_med14,mu_p15,delta_p16,theta_p17,omega_p18,beta_mh19,beta_mk20,&

!beta_mp21,beta_m2h22,beta_m2k23,beta_m2p24,delta_beta25,&

!theta_beta26

call odeparms(26, parms)

pi = 4.D0*atan(1.D0)

do i = 1, n

if (nm(i) >= parms(4)) then

Nm_mod(i) = parms(3) * nm(i)

else

Nm_mod(i) = parms(3) * parms(4)

endif

enddo

mu_m = -365.25 * log(0.9)

do i = 1, n-1

Nm_mod(i+1) = max(Nm_mod(i+1), Nm_mod(i)*exp(-mu_m*(years(i+1) - years(i))))

enddo

do i = 1, n

if (nm2(i) >= parms(5)) then

Nm2_mod(i) = parms(3) * nm2(i)

else

Nm2_mod(i) = parms(3) * parms(5)

endif

enddo

do i = 1, n-1

Nm2_mod(i+1) = max(Nm2_mod(i+1), Nm2_mod(i)*exp(-mu_m*(years(i+1) - years(i))))

enddo

bm(n) = 0.D0

bm2(n) = 0.D0

do i = 1, n-1

bm(i) = max(0.D0, (log(Nm_mod(i+1)) - log(Nm_mod(i)))/(years(i+1)-years(i))+mu_m)

bm2(i) = max(0.D0, (log(Nm2_mod(i+1)) - log(Nm2_mod(i)))/(years(i+1)-years(i))+mu_m)

enddo

bk(1:n) = parms(9) * (1.D0 + parms(10) * sin(2.D0*pi*(years(1:n) - parms(11))))

bp(1:n) = parms(15) * (1.D0 + parms(16) * sin(2.D0*pi*(years(1:n) - parms(17))))

Nk(1:n) = parms(8) * exp(-parms(10) * parms(9) * cos(2.D0*pi*(years(1:n)-parms(11))) / (2.D0*pi) )

Np(1:n) = parms(14) * exp(-parms(16) * parms(15) * cos(2.D0*pi*(years(1:n)-parms(17))) / (2.D0*pi) )

return

end

subroutine loadstuff(i, x)

integer i,n

double precision, dimension(5999) :: x, data

common /data/data,n

n = i

data = x

end

subroutine derivs (neq, t, y, ydot, yout, ip)

double precision t, pi

double precision dens,gamma_h,scale_m,Nm_min,Nm2_min,v,&

gamma_k,N_K_med,mu_k,delta_k,theta_k,omega_k,gamma_p,&

N_P_med,mu_p,delta_p,theta_p,omega_p,beta_mh,beta_mk,&

beta_mp,beta_m2h,beta_m2k,beta_m2p,delta_beta,&

theta_beta

double precision :: b_h, b_m, b_m2, b_k, b_p, N_m, N_m2, N_k, N_p, mu_m

integer neq, index, i, n, ip(*)

double precision, dimension(8) :: y, ydot

double precision, dimension(*) :: yout

double precision, dimension(1500) :: bm,bm2,bk,bp,Nm_mod,Nm2_mod,Nk,Np,Nh_mod,years,nm,nm2

double precision, dimension(1499) :: bh

common /myparms/dens,gamma_h,scale_m,Nm_min,Nm2_min,v,&

gamma_k,N_K_med,mu_k,delta_k,theta_k,omega_k,gamma_p,&

N_P_med,mu_p,delta_p,theta_p,omega_p,beta_mh,beta_mk,&

beta_mp,beta_m2h,beta_m2k,beta_m2p,delta_beta,&

theta_beta

common /data/years,nm,nm2,bh,n

common /derived/bm,bm2,bk,bp,Nm_mod,Nm2_mod,Nk,Np,Nh_mod

if(ip(1) < 1) call rexit("nout should be at least 1")

where (y < 0.D0) y = 0.D0

index = n-1

do i = 2, n

if (years(i) > t) then

index = i - 1

exit

endif

enddo

!index = findloc(years > t, .true.) - 1

b_h = bh(index)

b_m = bm(index)

b_m2 = bm2(index)

b_k = bk(index)

b_p = bp(index)

N_h = Nh_mod(index)

if (dens == 1.D0) then ! if density dependent, use analytic solution for population sizes

N_m = Nm_mod(index)

N_m2 = Nm2_mod(index)

N_k = Nk(index)

N_p = Np(index)

else ! otherwise assume all populations are size '1' despite them changing

N_m = 1.D0

N_m2 = 1.D0

N_k = 1.D0

N_p = 1.D0

endif

beta_mod = 1.D0 + delta_beta * sin(2.D0*pi*(t - theta_beta)) ! calculate current position in annual beta cycle

! actually calculate values

ydot = (/ &

beta_mod * (1.D0 - y(1)) * (beta_mk * y(2) * N_k + beta_mp * y(4) * N_p + beta_mh * y(6) * N_h) - y(1) * b_m * (1.D0 - v), &

beta_mod * (1.D0 - y(3) - y(2)) * (beta_mk * y(1) * N_m + beta_m2k * y(8) * N_m2) - (gamma_k + b_k) * y(2) + omega_k * y(3), &

gamma_k * y(2) - (b_k + omega_k) * y(3), &

beta_mod * (1.D0 - y(5) - y(4)) * (beta_mp * y(1) * N_m + beta_m2p * y(8) * N_m2) - (gamma_p + b_p) * y(4) + omega_p * y(5), &

gamma_p * y(4) - (b_p + omega_p) * y(5), &

beta_mod * (1.D0 - y(7) - y(6)) * (beta_mh * y(1) * N_m + beta_m2h * y(8) * N_m2) - (gamma_h + b_h) * y(6), &

gamma_h * y(6) - b_h * y(7), &

beta_mod * (1.D0 - y(8)) * (beta_m2k * y(2) * N_k + beta_m2p * y(4) * N_p + beta_m2h * y(6) * N_h) - y(8) * b_m2 * (1.D0 - v) &

/)

return

end

R code:

#Run

{

rm(list=ls())#clears any stored objects

library(lubridate)

library(rmutil)

library(caret)

filename = '20151214 peel scenarios.Rdata'

# load libraries

library(deSolve)

dyn.load('mortlimit.dll')

dyn.load("rrv.dll")

# load and process data

data = read.csv('Western Australia/FIMMWA modelling/Beeton modelling outcomes/Code and data/Peel merg-interp 90-13 for R.csv')

#Load data

{

sero.max = 0.75

data = na.omit(data)

years = strptime(data$Date, '%d/%m/%Y')

firstday = 1970 + as.numeric(min(years))/86400/365.25 # first date in data in decimal year format

years = as.numeric((years - min(years))/86400/365.25) # same for rest of them

data = data[order(years),] # order by date

years = years[order(years)]

ii = which(diff(years)>0) # remove same-day rows

data = data[ii,]

years = years[ii]

d.years = diff(years)

N.samples = nrow(data) # number of samples

Nm = data$Ae.camptorhynchus # mosquitoes pop data

Nm2 = data$Ae.vigilax

# scale to maximum of Ae campto

Nm2 = Nm2 / max(Nm)

Nm = Nm / max(Nm)

mu.m = -365.25 * log(1 - 0.1) # mortality rate of mosquitoes = 10%/day

mu.h = log(1 + 0.04) # human mortality (2% + another 2% for migration)

# piecewise linear interpolation human growth data

Nh = data$HumanPop # human pop data

Nh.mod = Nh

index.step = c(1,which(diff(Nh)>0)+1, N.samples+1) # work out points in time series for which human population increases

Nh.step = c(Nh[index.step[-length(index.step)]], Nh[N.samples]) # work out population at these points

years.step = c(years[index.step[-length(index.step)]], years[N.samples]) # work out time at these points

for (i in 1:N.samples) # go through Nh.mod

{

index = max(which(index.step <= i)) # find which step in population size we're at, and interpolate

Nh.mod[i] = Nh.step[index] + (years[i] - years.step[index]) / (years.step[index+1] - years.step[index]) * (Nh.step[index+1] - Nh.step[index])

}

bh = log(Nh.mod[-1]/Nh.mod[-N.samples])/diff(years) + mu.h # calculate human birth rate

L = length(years)

if (L>1500) stop('too many data points for Fortran code')

chunk = numeric(5999)

chunk[1:L] = years

chunk[1500+(1:L)] = Nm

chunk[3000+(1:L)] = Nm2

chunk[4500+(1:(L-1))] = bh

out = .Fortran('loadstuff', i = L, x = chunk)

}

#ODE Set up

{

dydt = function(t, y, parms)

{

y = pmax(y, 0) # make sure no value is negative, avoid crashes

im = y[1];

ik = y[2]; rk = y[3]

ip = y[4]; rp = y[5]

ih = y[6]; rh = y[7]

im2 = y[8]

index = max(which(years <= t)) # find where we are in the time series and extract relevant data - this GREATLY speeds up the model without discernible loss of accuracy

b.h = bh[index]

b.m = bm[index]

b.m2 = bm2[index]

b.k = bk[index]

b.p = bp[index]

N.h = Nh.mod[index]

if (parms$dens == TRUE) # if density dependent, use analytic solution for population sizes

{

N.m = Nm.mod[index]

N.m2 = Nm2.mod[index]

N.k = Nk[index]

N.p = Np[index]

}

else # otherwise assume all populations are size '1' despite them changing

{

N.m = 1

N.m2 = 1

N.k = 1

N.p = 1

}

beta.mod = 1 + parms$delta.beta * sin(2*pi*(t - parms$theta.beta)) # calculate current position in annual beta cycle

# actually calculate values

dydt = c(

b.m * (1-parms$v) * N.m + beta.mod * (parms$beta.mk * ((1 - im) * ik / N.m) + parms$beta.mp * ((1 - im) * ip / N.m) + parms$beta.mh * ((1 - im) * ih / N.m)) - im*b.m, #im

beta.mod * (parms$beta.mk * ((1 - rk - ik) * im * N.m) + parms$beta.m2k * ((1 - rk - ik) * im2 * N.m2)) - (parms$gamma.k + b.k) * ik + parms$omega.k * rk , #ik

parms$gamma.k * ik - (b.k + parms$omega.k) * rk, #rk

beta.mod * (parms$beta.mp * ((1 - rp - ip) *im * N.m) + parms$beta.m2p * ((1 - rp - ip) * im2 * N.m2)) - (parms$gamma.p + b.p) * ip + parms$omega.p * rp, #ip

parms$gamma.p * ip - (b.p + parms$omega.p) * rp, #rp

beta.mod * (parms$beta.mh * ((1 - rh - ih) *im * N.m) + parms$beta.m2h * ((1 - rh - ih) * im2 * N.m2)) - (parms$gamma.h + b.h) * ih, #ih

parms$gamma.h * ih - b.h * rh, #rh

b.m2 * (1-parms$v) * N.m2 + beta.mod * (parms$beta.m2k * ((1 - im2) * ik / N.m2) + parms$beta.m2p * ((1 - im2) * ip / N.m2) + parms$beta.m2h * ((1 - im2) * ih / N.m2)) - im2 * b.m2 #im2

)

list(dydt)

}

# Wrapper for running ODE model

model = function(y0, t.vec, dydt, params, rtol = 1e-3, ...)

{

Y1 = lsode(y0, t.vec, func = "derivs", dllname = "rrv", initfunc = "initmod",

parms = as.numeric(unlist(params)),

rtol = rtol, nout = 1)[,1:9]

Y1 # this is the output

}

#false positives only occur after 2006

past06 = years > (2006 - as.numeric(firstday))

ii = which(years > 1) # data after the first year

}

# function to plot data

{

plot.data = function(tofiles = FALSE, filename = 'default')

{

if (!tofiles)

dev.set(2)

else

pdf(file = paste(filename, 'rec.pdf', sep='_'))

par(cex=1.5, lwd=2)

subset = c(1,3,5,7,8)

matplot(as.numeric(firstday) + t.vec, 100*Y1[,1+subset], type='l', col=col.style[subset], lwd = lwd.style[subset], lty = lty.style[subset], xlab='Year', ylab='Proportion in class (%)',

log='', ylim = c(0, 100))

grid()

legend('right',

legend =

c('Ae. camp. prev.', 'Macropod prev.', 'Macropod rec.', 'Possum prev.', 'Possum rec.', 'Human prev.', 'Human rec.', 'Ae. vigilax prev.')[subset],

col=col.style[subset], lwd = lwd.style[subset], lty = lty.style[subset], bg='white', cex=0.5)

if (tofiles) dev.off()

if (!tofiles)

dev.set(3)

else

pdf(file = paste(filename, 'prev.pdf', sep='_'))

par(cex=1.5, lwd=2)

subset = c(2,4,6)

matplot(as.numeric(firstday) + t.vec, 100*Y1[,1+subset], type='l', col=col.style[subset], lwd = lwd.style[subset], lty = lty.style[subset], xlab='Year', ylab='Prevalence (%)',

log='y', ylim = c(1e-3, 100), axes = FALSE)

axis(1)

axis(2, at = 10^(-3:2), labels = prettyNum(10^(-3:2)))

box(bty = 'O')

grid()

legend('right',

legend =

c('Ae. camp. prev.', 'Macropod prev.', 'Macropod rec.', 'Possum prev.', 'Possum rec.', 'Human prev.', 'Human rec.', 'Ae. vigilax prev.')[subset],

col=col.style[subset], lwd = lwd.style[subset], lty = lty.style[subset], bg='white', cex=0.5)

if (tofiles) dev.off()

if (!tofiles)

dev.set(4)

else

pdf(file = paste(filename, 'fit.pdf', sep='_'), width = 14, height = 7)

par(cex=1.5, lwd=4)

plot(as.numeric(firstday) + years, data$pcRRV/100000*100, col='blue', lwd=3, type='o', pch=19, cex=0.5, ylim = c(0,scale), xlab = 'Years', ylab = 'Prevalence (%)')

# add in central 99% confidence intervals

cases.min = qbetabinom(0.005,

size = round(Nh.mod[ii]),

m = Y1[pmin(near.coords[ii], N.t), 7] * (1 - beta.g) + alpha.g * past06[ii],

s = s.g)/round(Nh.mod[ii])*100

cases.max = qbetabinom(0.995,

size = round(Nh.mod[ii]),

m = Y1[pmin(near.coords[ii], N.t), 7] * (1 - beta.g) + alpha.g * past06[ii],

s = s.g)/round(Nh.mod[ii])*100

polygon(as.numeric(firstday) + c(years[ii], rev(years[ii])), c(cases.min, rev(cases.max)), col='#FF000040', border = NA)

# adds in model result (of REPORTED prevalence via clinical notifications)

lines(as.numeric(firstday) + t.vec, 100*(Y1[,7] * (1 - beta.g) + alpha.g * past06.full), type='l', col='red', lty=1, lwd=4, xlab = 'Years', ylab = 'Prevalence (%)')

# works out mosquito population

Nm.mod = pmax(Nm*params$scale.m, params$Nm.min) # create new variable for mosq pop, to make sure population never drops below Nm.min

Nm.mod = .Fortran('mortlimit', L = length(Nm.mod), N = Nm.mod, d = d.years, mu = mu.m)$N

# plotting it

lines(as.numeric(firstday) + years, scale * Nm.mod/max(Nm.mod, na.rm=TRUE), lwd=3, col='dark green', lty=2)

# plots a vertical line for every year

abline(v=as.numeric(firstday) + 0:30, lty=2, col='grey', lwd=2)

legend('topright', legend = c('Modelled human reported prevalence', 'Actual human reported prevalence', 'Scaled mosquito abundance'), col=c('red', 'blue', 'dark green'), pch = c(NA, 19, NA), lwd = c(4,3,3), lty = c(1,1,2), bg='white', cex=0.7)

if (tofiles) dev.off()

}

}

# runs mosquito model AND WORKS OUT GOODNESS OF FIT (max like)

{

run.model = function(params, ...)

{

Y1 <<- model(y0, t.vec, dydt, params) # run model

if (nrow(Y1) == length(t.vec))# # all data present

{

if (!all(Y1[it,4]>sero.max) & !all(Y1[it,6]>sero.max) & all(Y1[it,8]<0.2)) # seroprevalence conditions on macropods and possums and humans

{

# function to work out likelihood given a certain false positive (alpha) and false negative (beta) rate

alphabeta = function(ab)

# function to work out likelihood given a certain false positive (alpha),

# false negative (beta) rate and overdispersion rate

sum(dbetabinom(data$RRV[ii], size = round(Nh.mod[ii]),

m = Y1[pmin(near.coords[ii], N.t), 7] * (1 - ab[2]) + ab[1] * past06[ii],

s = ab[3], log=TRUE))

#5-5-5 best

# finds best fitting false positives and negatives

O = optim(c(0,5,5), function(x) alphabeta(c(1/(1+10^x[1:2]), exp(x))), control = list(fnscale = -1))

res = O$value # max log-likelihood (for alpha and beta) (NOT AIC)

alpha.g <<- 1/(1+10^O$par[1])

beta.g <<- 1/(1+10^O$par[2])

s.g <<- exp(O$par[3])

#plot.data(...) # only plot data when well behaved

}

else

res = -Inf

}

else

res = -Inf

print(res)

res

}

run.model.nores = function(params, ...)

{

Y1 <<- model(y0, t.vec, dydt, params) # run model

if (nrow(Y1) == length(t.vec))# # all data present

{

# if (!all(Y1[it,4]>sero.max) & !all(Y1[it,6]>sero.max) & all(Y1[it,8]<0.2)) # seroprevalence conditions on macropods and possums and humans

# {

# function to work out likelihood given a certain false positive (alpha) and false negative (beta) rate

alphabeta = function(ab)

sum(dbetabinom(data$RRV[ii], size = round(Nh.mod[ii]),

m = Y1[pmin(near.coords[ii], N.t), 7] * (1 - ab[2]) + ab[1] * past06[ii],

s = ab[3], log=TRUE))

# finds best fitting false positives and negatives

O = optim(c(0,5,5), function(x) alphabeta(c(1/(1+10^x[1:2]), exp(x))), control = list(fnscale = -1))

res = O$value # max log-likelihood (for alpha and beta) (NOT AIC)

alpha.g <<- 1/(1+10^O$par[1])

beta.g <<- 1/(1+10^O$par[2])

s.g <<- exp(O$par[3])

#plot.data(...) # only plot data when well behaved

# }

# else

# res = -Inf

}

else

res = -Inf

print(res)

res

}

}

#Parameters

{

parms = list(

dens = T, # is it density dependent?

# human

gamma.h = x, # recovery rate

# mosquito

scale.m = x,

Nm.min = x, # minimum possible mosquito abundance

Nm2.min = x, # minimum possible mosquito abundance

v = x, # vertical transmission

# kangaroo

gamma.k = x, # recovery rate

N.K.med = x, # population size

mu.k = x, # mortality

delta.k = x, # seasonality

theta.k = x, # phase of kangaroo birth (best-fit estimate from data)

omega.k = x, # reinfection of recovered kangaroos

# possum

gamma.p = x, # recovery rate

N.P.med = x, #

mu.p = x, # mortality

delta.p = x, # seasonality

theta.p = x, # phase of possum birth (best-fit estimate from data)

omega.p = x, # reinfection of recovered kangaroos

# disease transmission

beta.mh = x, # mosq-human transmission rate

beta.mk = x, # mosq-kangaroo transmission rate

beta.mp = x, # mosq-possum transmission rate

beta.m2h = x, # mosq2-human transmission rate

beta.m2k = x, # mosq2-kangaroo transmission rate

beta.m2p = x, # mosq2-possum transmission rate

delta.beta = x, # seasonality

theta.beta = x # phase

)

}

# setup for running models

{

maxT = max(years) # maximum time required based on data

dt = 0.02763 # size of timestep (this seems to be stable)

t.vec = seq(0, maxT, dt) # vector of times to run over

N.t = length(t.vec) # length of vector

near.coords = round(years/dt + 1) # where each data point fits on our time vector

past06.full = t.vec > (2006 - as.numeric(firstday))

it = which(t.vec>1)

# setup for plots (colours, line widths and types)

col.style = c(rep('grey', 1), rep('orange', 2), rep('brown', 2), rep('red',2), 'black')

lwd.style = c(2,rep(3,6),2)

lty.style = c(1,rep(c(1,2),3),1)

subset = 1:8

# run model with current settings

t.start = proc.time()[3]

# im ik rk ip rp ih rh im2

#y0 = c(0.1, 0.02, 0.9, 0, 0, 0, 0.01, 0.1) # initial conditions

y0 = c(0.01, 0, 0, 0, 0, 0, 0, 0)

params = parms

x = c(-4, -0.3, -0, -0.3, 0.821, 0, 6, -6, -6) # putting in example parameters

x = c(1e-3 / (1 + 10^-x[1:3]), 10^x[4], x[5], 0.01 / (1 + 10^-x[6]), 1 + 10^x[7], 10^x[8:9], x[10]) # scaling them

}

##This code is from the initial models and figuring out what all the parameter values were##

############################################################################################

# moving on to optimisation

{

## Run for models

{

run.model.all = function(x, ...) # runs the model using the vector of free parameters

{

params = parms

params$beta.mh = x[1]; params$beta.mk = x[2]; params$beta.mp = x[3]

params$beta.m2h = x[4]*x[1]; params$beta.m2k = x[4]*x[2]; params$beta.m2p = x[4]*x[3]

params$delta.beta = x[10]; params$theta.beta = x[5]

params$Nm.min = x[6]; params$Nm2.min = x[6]; params$scale.m = x[7];

params$omega.k = x[8]; params$omega.p = x[9]

print(paste(x, collapse=', ')) # print out the parameter values each time

run.model(params, ...)

}

run.model.all.10 = function(x, ...) # this does scaling

{

res = run.model.all(c(1e-3 / (1 + 10^-x[1:3]), 10^x[4], pmax(pmin(x[5],1),0), pmax(0.01 / (1 + 10^-x[6]),0), 1 + 10^x[7], (1/(1+exp(-x[8:9]))), x[10]), ...)

x.mat <<- rbind(x.mat, x)

res.vec <<- c(res.vec, res)

res

}

# mh = mosquito/human transmission

# mk = mosuito/kangaroo transmission

# mp = mosquito/possum transmission

# m2 = scale of Ae. vigilax

# s.phase = phase of seasonality of transmission

# min = min proportion of mosquitoes

# max = max NUMBER of mosquitoes

# re.k = reinfection of kangaroos

# re.p = reinfection of possums

# s.amp = amplitude of seasonality of trasmission (either 0 or 1)

# mh mk mp m2 s.phase min max re.k re.p s.amp

x.def = c(-Inf, -Inf, -Inf, -Inf, 0, -Inf, -Inf, -Inf, -Inf, 0)

# runs model with only a subset of model components (vectors, hosts, seasonality etc)

run.model.subset.10 = function(y, ...)

{

x = x.def

if (10 %in% vars) # if seasonality, set amplitude straight to 1

{

x[10] = 1

vars = vars[vars != 10]

}

x[vars] = y

res = try(run.model.all.10(x, ...), silent = TRUE)

if (inherits(res, 'try-error')) res = -Inf

res

}

# initialises storage of model runs

x.mat = NULL

res.vec = NULL

nn=0

# these are each of the different model subsets

# this uses multiple different start values for the optimiser to find the best likelihoods

nn=nn+1

x.mh = rnorm(3)#c(0, 0, 0)

if (nn==1) y.init = x.mh else y.init = x.mh + rnorm(3)

vars = c(1, 6:7) # mh

while (run.model.subset.10(y.init) == -Inf) y.init = x.mh + rnorm(3)

O = optim(y.init, run.model.subset.10, control = list(fnscale = -1))

O = optim(O$par, run.model.subset.10, control = list(fnscale = -1))

res.mh = O$value

x.mh = O$par

x.mhk = rnorm(4)#c(0, 0, 0, 0)

if (nn==1) y.init = x.mhk else y.init = x.mhk + rnorm(4)

vars = c(1:2, 6:7) # mhk

while (run.model.subset.10(y.init) == -Inf) y.init = x.mhk + rnorm(4)

O = optim(y.init, run.model.subset.10, control = list(fnscale = -1))

O = optim(O$par, run.model.subset.10, control = list(fnscale = -1))

res.mhk = O$value

x.mhk = O$par

x.mhkp = rnorm(5)#rep(0, 5)

if (nn==1) y.init = x.mhkp else y.init = x.mhkp + rnorm(5)

vars = c(1:3, 6:7) # mhkp

while (run.model.subset.10(y.init) == -Inf) y.init = x.mhkp + rnorm(5)

O = optim(y.init, run.model.subset.10, control = list(fnscale = -1))

O = optim(O$par, run.model.subset.10, control = list(fnscale = -1))

res.mhkp = O$value

x.mhkp = O$par

x.mhks = rnorm(5)#rep(0, 5)

if (nn==1) y.init = x.mhks else y.init = x.mhks + rnorm(5)

vars = c(1:2, 5:7, 10) # mhks

while (run.model.subset.10(y.init) == -Inf) y.init = x.mhks + rnorm(5)

O = optim(y.init, run.model.subset.10, control = list(fnscale = -1))

O = optim(O$par, run.model.subset.10, control = list(fnscale = -1))

res.mhks = O$value

x.mhks = O$par

x.mhkps = rnorm(6)#rep(0, 6)

if (nn==1) y.init = x.mhkps else y.init = x.mhkps + rnorm(6) # there was an error

vars = c(1:3, 5:7, 10) # mhkps

while (run.model.subset.10(y.init) == -Inf) y.init = x.mhkps + rnorm(6)

O = optim(y.init, run.model.subset.10, control = list(fnscale = -1))

O = optim(O$par, run.model.subset.10, control = list(fnscale = -1))

res.mhkps = O$value

x.mhkps = O$par

x.mhkpr = rnorm(7)#rep(0, 7)

if (nn==1) y.init = x.mhkpr else y.init = x.mhkpr + rnorm(7)

vars = c(1:3, 6:9) # mhkpr

while (run.model.subset.10(y.init) == -Inf) y.init = x.mhkpr + rnorm(7)

O = optim(y.init, run.model.subset.10, control = list(fnscale = -1))

O = optim(O$par, run.model.subset.10, control = list(fnscale = -1))

res.mhkpr = O$value

x.mhkpr = O$par

x.mhkprs = rnorm(8)#rep(0, 8)

if (nn==1) y.init = x.mhkprs else y.init = x.mhkprs + rnorm(8)

vars = c(1:3, 5:10) # mhkprs

while (run.model.subset.10(y.init) == -Inf) y.init = x.mhkprs + rnorm(8) # there was an error

O = optim(y.init, run.model.subset.10, control = list(fnscale = -1))

O = optim(O$par, run.model.subset.10, control = list(fnscale = -1))

res.mhkprs = O$value

x.mhkprs = O$par

x.mmhk = rnorm(5)#c(0, 0, 0, 0, 0)

if (nn==1) y.init = x.mmhk else y.init = x.mmhk + rnorm(5)

vars = c(1:2, 4, 6:7) # mmhk

while (run.model.subset.10(y.init) == -Inf) y.init = x.mmhk + rnorm(5)

O = optim(y.init, run.model.subset.10, control = list(fnscale = -1))

O = optim(O$par, run.model.subset.10, control = list(fnscale = -1))

res.mmhk = O$value

x.mmhk = O$par

x.mmhkp = rnorm(6)#rep(0, 6)

if (nn==1) y.init = x.mmhkp else y.init = x.mmhkp + rnorm(6)

vars = c(1:4, 6:7) # mmhkp

while (run.model.subset.10(y.init) == -Inf) y.init = x.mmhkp + rnorm(6)

O = optim(y.init, run.model.subset.10, control = list(fnscale = -1))

O = optim(O$par, run.model.subset.10, control = list(fnscale = -1))

res.mmhkp = O$value

x.mmhkp = O$par

x.mmhkps = rnorm(7)#rep(0, 7)

if (nn==1) y.init = x.mmhkps else y.init = x.mmhkps + rnorm(7)

vars = c(1:7, 10) # mmhkps

while (run.model.subset.10(y.init) == -Inf) y.init = x.mmhkps + rnorm(7)

O = optim(y.init, run.model.subset.10, control = list(fnscale = -1))

O = optim(O$par, run.model.subset.10, control = list(fnscale = -1))

res.mmhkps = O$value

x.mmhkps = O$par

x.all = rnorm(9)#rep(0,9)

if (nn==1) y.init = x.all else y.init = x.all + rnorm(9)

vars = c(1:10) # all

while (run.model.subset.10(y.init) == -Inf) y.init = x.all + rnorm(9)

O = optim(y.init, run.model.subset.10, control = list(fnscale = -1))

O = optim(O$par, run.model.subset.10, control = list(fnscale = -1))

res.all = O$value

x.all = O$par

save.image(filename)

#stop('stop')

max.LL = matrix(NA, 7, 11)

max.parms = array(NA, c(7, 11, 10))

for (j in 1:7)

{

code = (x.mat != outer(rep(0, nrow(x.mat)), x.def, FUN = '+')) %*% 2^(9:0)

table(code)

codes = c(536, 792, 920, 825, 953, 926, 959, 856, 984, 1017, 1023)

codenames = c('mh','mhk','mhkp','mhks','mhkps','mhkpr','mhkprs','mmhk','mmhkp','mmhkps','all')

# dev.new()

for (i in 1:length(codes))

{

print(codenames[i])

ss = which(code == codes[i])

if (length(ss)>0)

{

best = max(res.vec[ss])

wbest = which(res.vec[ss] == best)[1]

x = x.mat[ss[wbest],]

print(x)

print(best)

y = c(1e-3 / (1 + 10^-x[1:3]), 10^x[4],pmax(pmin(x[5],1),0), pmax(0.01 / (1 + 10^-x[6]),0), 1 + 10^x[7], (1/(1+exp(-x[8:9]))), x[10])

run.model.all(y, tofiles = TRUE, filename = paste(codenames[i], "Name", sep="_"))

max.LL[j, i] = best

max.parms[j, i, ] = y

dev.set(5);

#plot(res.vec[ss]); abline(v = wbest, lty=2, col='red')

out = -log(-(res.vec[ss]-best))

#plot(out, axes = FALSE)

#axis(1); axis(2, at = pretty(out, n=10), labels = round(best - exp(- pretty(out, n=10)), digits = 2), las = 1)

#abline(v = wbest, lty=2, col='red'); abline(h = -log(-(-1850 - best)), lty=2, col='blue')

}

print('')

}

}

bestruns = apply(max.LL, 2, function(x) order(x)[7])

best.LL = max.LL[cbind(bestruns, 1:11)]

best.parms = matrix(NA, 11, 10)

for (i in 1:11) best.parms[i,] = max.parms[bestruns[i], i, ]

for (i in 1:11)

{

print(codenames[i])

y = best.parms[i,]

run.model.all(y, tofiles = TRUE, filename = paste(codenames[i], "Run", sep="_"))

}

df = data.frame(best.LL, best.parms)

rownames(df) = codenames

colnames(df) = c(

"maximum log-likelihood",

"mosquito/human transmission",

"mosquito/kangaroo transmission",

"mosquito/possum transmission",

"scale of Ae. vigilax",

"phase of seasonality of transmission",

"min proportion of mosquitoes",

"max NUMBER of mosquitoes",

"reinfection of kangaroos",

"reinfection of possums",

"amplitude of seasonality of trasmission (either 0 or 1)"

)

}

}

}
