# Supplementary material for: Testing the intrinsic mechanisms driving the dynamics of Ross River Virus across Australia
Source: PLoS Pathog. 2024 Feb 15;20(2):e1011944. doi: 10.1371/journal.ppat.1011944 (PMC10868856; doi:10.1371/journal.ppat.1011944)
Supplement: S3 Appendix — Transmission scenarios model combinations for explaining RRV transmission across epidemic centres in Australia. (DOCX) [file ppat.1011944.s003.docx]

Sup Table 1: Transmission scenarios model combinations for explaining RRV transmission across epidemic centres in Australia.

| Scenario | $\boldsymbol{\beta}_{\boldsymbol{vh}}$ | $\boldsymbol{\beta}_{\boldsymbol{vk}}$ | $\boldsymbol{\beta}_{\boldsymbol{vp}}$ | ***v^2^*** | $\boldsymbol{\beta}_{\boldsymbol{\mu}}$ | ***v_min_*** | ***v_max_*** | $\boldsymbol{\omega}_{\boldsymbol{k}}$ | $\boldsymbol{\omega}_{\boldsymbol{p}}$ | $\boldsymbol{\beta}_{\boldsymbol{\varphi}}$ |
| --- | --- | --- | --- | --- | --- | --- | --- | --- | --- | --- |
| 1 | X |  |  |  |  | Yes | Yes |  |  |  |
| 2 | X | X |  |  |  | Yes | Yes |  |  |  |
| 3 | X | X | X |  |  | Yes | Yes |  |  |  |
| 4 | X | X | X |  |  | Yes | Yes | X | X |  |
| 5 | X | X | X |  | X | Yes | Yes | X | X | X |
| 6 | X | X | X |  | X | Yes | Yes |  |  | X |
| 7 | X | X |  |  | X | Yes | Yes |  |  | X |
| 8 | X | X |  | X |  | Yes | Yes |  |  |  |
| 9 | X | X | X | X |  | Yes | Yes |  |  |  |
| 10 | X | X | X | X | X | Yes | Yes |  |  | X |
| 11 | X | X | X | X | X | Yes | Yes | X | X | X |
