# Supplementary material for: Testing the intrinsic mechanisms driving the dynamics of Ross River Virus across Australia
Source: PLoS Pathog. 2024 Feb 15;20(2):e1011944. doi: 10.1371/journal.ppat.1011944 (PMC10868856; doi:10.1371/journal.ppat.1011944)
Supplement: Attachment — Submitted filename: Response to reviewers.docx [file ppat.1011944.s004.docx]

**Comments from the editors and reviewers:**

**Part I - Summary**
Please use this section to discuss strengths/weaknesses of study, novelty/significance, general execution and scholarship.
Reviewer #1: I have reviewed he manuscript titled “Testing the intrinsic mechanisms driving the dynamics of Ross River Virus across Australia” submitted to PLOS Pathogens.
The manuscript describes the use of modelling to determine the mechanisms most likely driving activity of mosquito-borne Ross River virus in Australia with an focus on regions where disease is often greatest. Investigations of this nature are particularly useful to local health authorities in developing response strategies to this and other mosquito-borne diseases.
Firstly, it would be a useful addition to the manuscript to add a brief description of the clinical aspects of the disease. While there are many papers on the topic, it would be beneficial here for readers unfamiliar with the disease. I note that there are already papers cited by authors that include clinical aspects. The brief descriptions could be added to second paragraph of Introduction.

**Response:**

**We have added in a description of the clinical features of the disease within the introduction (Lines 83-87)**

Reviewer #2: I very much appreciate the aims of the paper. I also appreciate the approach taken by comparing a set of models of increasing complexity. However, not having models within the set of alternative models that account for interannual variation makes little sense to me within the Australian context. If there is one thing characteristic for candidate RRV vector and notably host species, it is their opportunistic life histories, resulting in (very) large population changes varying in concert with the erratic climatic conditions on the Australian continent. Fluctuations that may supersede any seasonal variations. There is a wealth of literature on (arbo) virus dynamics on the Australian continent that illustrate these multi-year dynamics.
I am also slightly disappointed in the learnings that we can take out of the presented modelling exercise. Authors claim that “The information gained here can be used to improve the management of disease dynamics beneficial in reducing health and economic impacts”, without providing clear avenues on how exactly this research could be used to that effect; this is entirely left to the creativity of the reader.

I concur with the authors that there is a paucity of information on the composition of the RRV reservoir and vector communities and on the role individual members of those communities might have in RRV dynamics. This almost inevitably means that it will be difficult to make predictive models. However, in the absence of accurate data, mathematical models can still be very valuable to identify the most crucial gaps and help prioritise empirical research and monitoring. Unfortunately, I failed to discover such guidance for future research in this manuscript.

**Response:**

**We thank the reviewer for their thoughtful feedback and agree that we have not been clear enough about future research directions. This was partially a deliberate strategy to not be too prescriptive or critical toward state, territory and local government area councils that invest significant efforts into their differing approaches and strategies to vector and disease risk management. However, we agree with the reviewer that we have been too tentative. Accordingly, we have pointed out in the abstract that we discuss future research and management directions, which can be found on lines 49-52**

**The reviewer also correctly points out well known extreme fluctuations in vector populations (which may also occur in reservoir hosts). While we have limited information on the extent of fluctuations in host population dynamics (see below), we note that our models are forced with vector surveillance data from each site (see lines 180-181) and thus accommodate this critical information. We have made revisions to make the vector-forcing information clearer throughout the manuscript, as the reviewers’ feedback suggests that this was not sufficiently evident.**

**Regarding the fluctuation in host populations, the reviewer is absolutely correct about current limitations, and accordingly we have acknowledged this as a leading limitation of our and others research in this area (e.g., see lines 644-647). Currently, continuous reservoir host population data in proximity to our study locations is extremely limited and thus our study has tried to make the most of what host-reservoir population data can be pulled together to inform mechanisms. Taking on-board the reviewers' recommendations we have expanded our discussion, pointing to the value of future site-specific host monitoring programs to improve mechanistic understanding of RRV dynamics going forward (see lines 649-655).**

Reviewer #3: This manuscript presents results of mechanistic SIR models fitted to Ross River Virus human notification data, with ancillary data from vector surveillance programs and wildlife abundance estimates used to investigate the support for different levels of transmission complexity across several Australian RRV epicentres. The study is nicely written and presented, though I am not an epidemiologist so cannot comment on the validity of the differential equations. However I do have some comments that I feel can improve the clarity and presentation of the work, so I have listed them below.

**Response:**

**We thank the reviewer for their valuable feedback.**

**Part II – Major Issues: Key Experiments Required for Acceptance**
Please use this section to detail the key new experiments or modifications of existing experiments that should be absolutely required to validate study conclusions.

Generally, there should be no more than 3 such required experiments or major modifications for a "Major Revision" recommendation. If more than 3 experiments are necessary to validate the study conclusions, then you are encouraged to recommend "Reject".

Reviewer #1: I have no comments on major issues associated with the paper.

Reviewer #2: “The primary aims of this study are to (1) disentangle key mechanisms responsible for driving observed human RRV dynamics; (2) estimate key parameters associated with the transmission of RRV and reporting of RRV notifications; and (3) assess how mechanisms driving RRV dynamics vary, or are similar, among epidemic sites around Australia.” Yet, how can these be investigated when there are such uncertainties regarding hosts and vector species and dynamics? Instead, I suggest focussing on conducting a sensitivity analysis to identify the most crucial model parameters that warrant further empirical research.
Also see Part I

**We appreciate the concerns raised by the reviewer, which we are happy to address. A strength of our study is that we have a reasonable degree of confidence for many parameters in our models (see Tables 1 & 2 and Methods text) and we estimate parameters that we are uncertain about through fitting our models to empirical human RRV notification data. With regard to vector and host species and dynamics, as mentioned above, our models are forced with empirical vector surveillance data, so these dynamics are explicitly accounted for. We are generally confident that we have accounted for the most important vectors species at each of our sites, and this is agreed upon by the many expert co-authors on the manuscript, although we also acknowledge that additional vectors may play a role (see lines 263-650 & 661-662). We believe that we have managed to approximate host dynamics relatively well, although we are clear about uncertainties there and that other hosts could be involved. Indeed, it is necessary to start with a reasonably accepted group of putative reservoir hosts to begin assessing their mechanistic role in human RRV notifications.**

**We also wonder if what we mean in Aim 3 by “assess how mechanisms driving dynamics vary” was not entirely clear to the reviewer. The way that this is addressed in this study is by explicitly testing different mechanistic hypotheses (models), parameterised with available empirical data, fit to empirical human RRV case data, and estimating what is unknown. This approach is advantageous for addressing Aim 3 because the likelihood of different mechanisms driving observed RRV dynamics can be explicitly evaluated among the hypotheses, and by replicating this across multiple sites we develop an understanding of mechanisms that are relatively consistent or variable. In a way, this gets at some to the uncertainties that the reviewer is justifiably concerned about. If the reviewer feels we have not be sufficiently clear about this, we would be happy to receive additional feedback on where that could be clarified.**

**In agreement with the reviewer, we are also very supportive of sensitivity analyses, particularly where specific parameters are uncertain. Indeed, a key outcome of this study is the value of future research conducting sensitivity analyses on some key parameters, particularly: host population dynamics to determine the impact that varying these can have on how the models fit to RRV notifications in humans (see lines 624-628); and in the dynamics of vector populations (e.g., timing and peak abundance) to assess how many human RRV cases are potentially being averted by existing vector management practices occurring across out study sites (see lines 628-630). In accordance with the reviewers’ feedback, we have increased emphasis on the value of future sensitivity analyses.**

Reviewer #3: (No Response)

**Part III – Minor Issues: Editorial and Data Presentation Modifications**
Please use this section for editorial suggestions as well as relatively minor modifications of existing data that would enhance clarity.

Reviewer #1: It may be worth noting in the description of study sites within Methods section that the eight locations included in study are all likely to have different communities of mosquitoes driving RRV transmission. As a consequence, climatic drivers of mosquito abundance will also differ. I note this is discussed later in manuscript but a state here would be useful. There is also a need to clearly state that the four mosquito species selected all vary greatly in their population dynamics, habitat associated and, to some degree, their host feeding preferences as currently known. Perhaps a brief statement on these issues could be included in the “vector monitoring and competence data” section of Methods would be useful.

**Thank you for the suggestions to add more context about the differences in vector ecology and dynamics across the study sites. Here we have made amendments in the Study Sites section (lines 193-195) and in the Vector Monitoring section (lines 259-263).**

The authors do include a comment about the potential for birds to be involved as reservoir hosts. It would be good to clarify in methods why bird density, or some other measure of bird diversity, was included. I am not proposing that it should have been included in analysis but a clear statement why it wasn’t would be useful.

**We appreciate the reviewer's suggestion to clarify why alternative host (including birds) density data were not included in our analysis. We have added a statement in the Methods section where reservoir hosts are introduced (lines 302-306 & 661-662, 644-647)**

**This statement clarifies that marsupial reservoir hosts were used as they have generally been discussed at the 'most likely' reservoirs in the literature, and we also were able to identify reasonable population approximations of species potentially involved in transmission. However, we acknowledge that other host species, such as birds and some placental mammals, may also have a role. While beyond the scope of this investigation, testing models that include alternative reservoir hosts in future studies would be valuable.**

In interpreting the results, I would be interested in the authors’ analysis of importance of having multiple vectors and differences in their relative abundance at each location. For example, some inland areas would only have two species present while other would have multiple (perhaps all four). Seasonality of species will also vary with species overlapping in temporal abundance while others less so. Does the analysis reveal the key mosquito species at each location?

**We appreciate the thoughtful suggestions on delving deeper into the implications of having multiple vector species present at each location. To address this within the constraints of our current model structure, we have added the following paragraph to the Results section (lines 473-481) and in the Discussion section (lines 553-556).**

**“All of our top models (as ranked by AIC) contain the two most numerically abundant vector species at each site suggesting an important role of both for human RRV (see Tables 1 and 4). While the incorporation of more complex vector communities (greater than two vector species) was beyond the scope of this investigation, addition of models containing more putative vector species would be valuable to better understand the critical vector community at each site.”**

**We have also added a statement in the Discussion calling for future work to expand the vector species analyzed to provide greater resolution into species-specific roles in RRV transmission.**

It would be interesting to see the authors comment on the potential differences should individual macropod species be isolated in analysis. For example, while there is a paucity of information about the specific role of species in RRV transmission, could there be differences related to population dynamics of different macropod species or groups, such as wallabies or kangaroos? There is some discussion of host contributions from the analysis but this point is of interest.

**We appreciate the reviewer raising the relevant point about potential differences among macropod species. We have accommodated differences among macropod species based on density, viremic period, and seasonality in births and, as the reviewer notes, these could all contribute to differences in RRV dynamics. Accordingly, we have expanded our discussion on variation among macropod species in these parameters and the potential role this could have in RRV dynamics among sites (see lines 632-642).**

A final minor comment. Authors should check journal requirements for abbreviation of scientific names. Also, a review of format of references cited is required to ensure consistency.

**Thank you for the response, we have updated the in-text figure referencing to be consistent with the journal. Upon follow up to this comment, the corresponding author is having difficulty with their citation manager to correct the references. In order to get this revision back in a timely matter, the reference section still have some minor formatting to be completed.**

Reviewer #2: The abstract was rather vague on what was researched and what the results are. Could be much more explicit. Examples: “major epidemic centres in Australia” , “We considered the importance of up to two vector species”, “two non-human reservoir host species”, “We found the combination of most important mechanisms to be relatively similar..”.

**Thank you for sharing this suggestion to improve the abstract. Accordingly, we have revised the abstract to make the methods and findings more explicit:**

**Ross River virus (RRV) is Australia’s most important mosquito-borne disease, with variable transmission dynamics across geographic regions. We used deterministic models to test mechanisms driving RRV dynamics across major epidemic centers in Brisbane, Darwin, Mandurah, Mildura, Gippsland, Renmark, Murray Bridge, and Coorong. We considered models with up to two vector species (Aedes vigilax, Culex annulirostris, Aedes camptorhynchus, Culex globocoxitus), two reservoir hosts (macropods, possums), seasonal transmission effects, and transmission parameters. We fit models against long-term RRV surveillance data (1991-2017) and used Akaike Information Criterion to select important mechanisms. The combination of two vector species, two reservoir hosts, and seasonal transmission effects explained RRV dynamics best across sites. Estimated vector-human transmission rate (average β=8.04x10^-4) was similar despite different dynamics. Models estimate 43% underreporting of RRV infections. Findings enhance understanding of RRV transmission mechanisms, provide parameter estimates to inform public health, and offer a basis to evaluate mitigation practices.**

Table 1: reproduction in Darwin being considered non-seasonal requires better justification.

**Agreed we have added in a sentence in the methods with a justification (Lines 321-323) and in the discussion (604-606).**

Line 166: three host species. Should this not be two?

**In the SIR context, humans are considered the third host species which is show in the referenced equation 1.**

That non-seasonal environmental forcing is lacking from the models (e.g. by assuming population dynamics of both vectors and hosts to be invariable across years) possibly explains why notably “modelled RRV notifications in Mildura, Gippsland, and Coorong were captured least well, compared to the other sites in non-outbreak periods (Table 5).”

**Our models was forced on empirical vector data (Lines 180-181), and also included seasonality in host reproduction (Table 1, Lines 321-331). It is possible that the reason these sites were captured least well was owing to limitations in host population dynamic data, possibly there may be greater complexity in the vector and host community involved in RRV transmission, and it is possible that the relatively fewer RRV cases in these inland sites may make them more difficult to capture the mechanisms driving dynamics.**

Reviewer #3: Line 84: “effects of climate on mosquito species communities”. Can you be more specific about what kinds of effects, i.e. on community composition, relative abundance, seasonal dynamics etc…

**We have updated this sentence to be more specific, discussing that climate can alter the species composition, species abundance, and alterations in the most abundant vector species.**

Line 121: SIR needs to be defined here

**Thank you. We have included this change.**

Line 210: I find it strange that the data descriptions are interrupted by the description of the model structure. To me it would make more sense to define the model first, and then describe all data that were used for modelling (i.e. move the sections about study location selection down below the model definition)

**Adjustment in the structure has been made as suggested.**

Line 225: How many observations of mosquito surveillance data were missing?

**This varies among the sites. We thank the reviewer for this question, as it has caused us to evaluate if we had presented the reader with sufficient information on vector surveillance, which we feel can be improved. Accordingly, we have added graphs to the supplementary material showing the surveillance for the two most abundance vectors for each site (lines 258-259), as well as our splines to estimate missing data where it did occur. We have also expanded our methods to cover vector surveillance differences among sites a little better and expanded our discussion to include this important context in interpretation of our results.**

Line 357: There is some discrepancy in the original aims of this work and the methods used to validate models. The validation procedure asks whether models fitted to the observed data can reliably reproduce known ‘outbreaks’, but the objective is defined as “obtain the most in-depth mechanistic understanding of transmission dynamics driving patterns of RRV incidence”. I appreciate that a custom model checking function was used, which is a good step to ensuring the model can be useful for a specific purpose, but a better description of the objectives would help to clarify the actual purpose of the model.

**We think the reviewer for this fair point and, accordingly have modified our objective and aims in the introduction to better align with the methods used:**

**“In this study, we bring together, to the best of our knowledge, the most extensive and diverse data on a vector-borne disease in Australia to assess the relative potential of hypothesised transmission mechanisms influencing the dynamics of RRV incidence.”**

**“The primary aims of this study are to (1) evaluate the relative likelihood of hypothesised mechanisms responsible for observed human RRV dynamics; (2) estimate key parameters associated with the transmission of RRV and reporting of RRV notifications; and (3) assess how hypothesised mechanisms driving RRV dynamics vary, or are similar, among epidemic sites around Australia.”**

On the same topic, wouldn’t it be more useful to compare models based on their out of sample forecast performances? I imagine this is our ultimate reason for employing such a model, to estimate the current state of the system given all available data up to today and then simulate forward trajectories to provide point-based or probabilistic predictions about the likelihood of outbreaks in the coming weeks / months. AIC based on in-sample simulations may not come close to giving a reliable picture of whether these models could be useful for this purpose because estimates of outbreaks were obtained using all data (before the outbreak and after). Could the authors consider implementing a cross-validation exercise to better scrutinise models on their predictive capacities? Or at least provide details describing why they do not think this would be insightful / necessary?

**Thank you. We have actually been quite deliberate in avoiding suggestions of forecasting. The goal of this work is about trying to help understand hypothesised mechanisms potentially shaping RRV incidence, which has been a long-standing issue in this system. We think ranking the different models with AIC and estimating parameters is therefore appropriate in our circumstance, although we are open to other suggestions if the reviewer still disagrees. Perhaps we had still not been clear enough about the scope of our study, so we have attempted to make this scope clearer in the introduction (lines 127-138) and discussion (lines 666-676).**

**It is possible that future work might look to use a similar mechanistic modelling approach for forecasting (in addition to statistical approaches already used), but that would likely need to be after more mechanisms have been explored and a mechanistic basis more settled upon. We have expanded our discussions to also make this point clearer (see lines 676-683).**

Line 364: It would be much easier to understand the workflow and comment on the analyses if the R code was provided. I realize data cannot be provided, but the code can still be more informative about any decisions that were made than the actual descriptions in the manuscript are. For example, I’m confused about how the PVO and NVO values were calculated. I understand how the observed outbreak values were calculated, but how were the predicted values calculated? Also, did these calculations consider the nonindependence of successive weeks (i.e. if week 30 is an ‘outbreak’ week, wouldn’t week 31 be more likely to also be an outbreak week)?

**Thank you. We have included a link to a version of the Fortran solver and a general R code used in the Supplementary Material. This code includes fitting of Scenario 11, parameter estimation, the calculation of PVO and NVO. The AIC rankings where done in post-processing in Microsoft's Excel, references 70 and 71 provide examples of how this is done.**

**To clarify, PVO and NVO are the same as standard Positive Predictive Value and Negative Predictive Value calculations (see below diagram), but we used V (validated) so as to more correctly capture the classification of outbreak or not. We have further clarified this in our methods (see lines 389-392).**

**Lastly, we considered at length about non-independence in the data in calculating PVO/NVO and ultimately decided to keep these calculations as simple as possible by analysing the full dataset. We think the results are a fair representation of how well the models capture outbreaks, although are open to different calculations if the reviewer feels what we have done is problematic.**

Taken from: <https://step1.medbullets.com/stats/101006/testing-and-screening>

Figure 2: These plots are hard to decipher, especially since the predictions from the models seem to be cut off by the upper y-axis limit in several plots.

**Thank you for the suggestion, we agree for many of the modelling peaks, these far exceed the observed notifications. Adjusting the y-axis to the maximum observed value makes the other lower notifications and model fit hard to observe. To balance this, we have split the figures up into groups of 4 to make them larger and easier to see.**

Table 6: It would be useful to report uncertainties of these parameters so any future studies that plan to use them can include more realistic assumptions

**Thank you for the suggestion to report uncertainties for the parameter estimates in Table 6. Unfortunately, because our model is a deterministic ODE model that assumes fixed parameter values, we did not generate measures of uncertainty such as confidence intervals or standard errors for the estimates.**

**Deterministic ODE models make point estimates of parameters by fitting to data, but do not characterise the uncertainty in those estimates. To quantify parameter uncertainty, we would need to use a statistical model fit in a Bayesian or likelihood framework that estimates a posterior distribution for each parameter. This type of analysis was outside the scope of our research aims and is more suited as an independent piece of research given its complexity.**

**Since we do not have uncertainty ranges from our current modelling approach, reporting confidence intervals would imply a falsely high degree of certainty in the estimates. I have included a statement on this limitation in the discussion on lines (632-642).**

Line 466: The findings of high probable underreporting is an important one that also raises questions of how best to validate such models. Do we want models that give an estimate of the true number of disease occurrences, even though they may be different to what is actually observed, or do we want models that can forecast number of notifications to help with resource planning? These are challenging questions but they deserve some attention

**This is a great point. As mentioned above, forecasting is not a goal of this study, but trying to construct models that represent the essential features that we understand about the system is. In that sense, we think it is appropriate to estimate an underreporting rate into the models we test, because RRV is well known to be underreported in humans, but the extent of underreporting is uncertain.**

**To date, there has been very little modelling exploration the extent of underreporting of RRV, and our modelling can at least begin to improve knowledge on the topic. This also has applied value, as health agencies are interested in both total likely exposures and symptomatic exposures. Furthermore, future research simulating the effectiveness of vector management can address both the total number of RRV cases averted as well as symptomatic cases..**

**In accordance with the reviewers’ suggestions, we have included a new discussion paragraph to specifically address this (lines 666-683).**
